# Receptor for advanced glycation end-products and child neglect in mice: A possible link to postpartum depression

**DOI:** 10.1016/j.cpnec.2022.100146

**Published:** 2022-06-03

**Authors:** Haruhiro Higashida, Maria Gerasimenko, Yasuhiko Yamamoto

**Affiliations:** a Department of Basic Research on Social Recognition and Memory, Research Center for Child Mental Development, Kanazawa University, Kanazawa, 920-8640, Japan; b Laboratory for Social Brain Studies, Research Institute of Molecular Medicine and Pathobiochemistry, And Department of Biochemistry, Krasnoyarsk State Medical University Named After Prof. V. F. Voino-Yasentsky, Krasnoyarsk, 660022, Russia; c Department of Biochemistry and Molecular Vascular Biology, Kanazawa University Graduate School of Medical Sciences, Kanazawa, 920-8640, Japan

**Keywords:** RAGE, Maternal behavior, Oxytocin, Stress, Postpartum, Peripartum, Puerperium

## Abstract

The receptor for advanced glycation end-products (RAGE), a pattern recognition molecule, has a role in the remodeling of vascular endothelial cells mainly in lungs, kidney and brain under pathological conditions. We recently discovered that RAGE binds oxytocin (OT) and transports it to the brain from circulation on neurovascular endothelial cells. We produced knockout mice of the mouse homologue of the human RAGE gene, *Ager*, designated RAGE KO mice. In RAGE KO mice, while hyperactivity has been reported in male mice, maternal behavior was impaired in female mice. After an additional stress, deficits in pup care were observed in RAGE KO mother mice. This resulted in pup death within 1–2 days, suggesting that RAGE plays a critical role during the postpartum period. Thus, RAGE seems to be important in the manifestation of normal maternal behavior in dams. In this review, we summarize the significance of brain OT transport by RAGE and propose that RAGE-dependent OT can dampen stress signals during pregnancy, delivery and early postpartum periods. To the best of our knowledge, there have been no previous articles on these RAGE-dependent results. Based on these results in mice, we discuss a potential critical role of RAGE in emotion swings at the puerperium (peripartum) and postpartum periods in women.

## Highlights

• RAGE play a role in oxytocin transport via the blood-brain barrier into the brain.
• RAGE KO dams had maternal behavior impairment after stress exposure that is in line with the two-hit theory. The first hit is RAGE signaling absence, the second hit is a stress event occurred in the postpartum period.
• We can hypothesize that RAGE signaling can affect maternal depression development through oxytocin transportation into the brain.

## 1 Introduction

New mothers sometimes experience postpartum ‘baby blues’ after childbirth [[1], [2], [3]]. “Baby blues” begin in the first two to three days after delivery and continues for up to two weeks. However, other new mothers experience a more severe and prolonged form of depression [2], known as postpartum depression [4,5]. Postpartum depression affects between 10% and 20% of new mothers, leading to mood swings, crying spells, anxiety, and difficulty in sleeping [6,7]. These conditions lead to higher health care costs and productivity loss. In addition, postpartum depression can affect infant care [63] that can result in lower breastfeeding rates [8], substantial impairment of mother-child bonding, and lower adherence to infant safety behaviors [9,10]. This may leave a lasting negative effect on the infant's cognitive and social development [[11], [12], [13]].

Reproductive hormonal changes may be pathophysiological in postpartum depression [14,15]. Oxytocin (OT) is one such reproductive hormone [[16], [17], [18], [19], [20], [21], [22], [23]]. In humans, it is known that blood OT concentrations remain high from the first to the last trimester as well as in the first postpartum month [[23], [24], [25]]. High OT levels are thought to be associated with maternal behaviors, such as mutual gaze, infant vocalization, affiliative touch, and frequent checks on the infant [17,24]. Therefore, throughout pregnancy and in the peripartum or postpartum period, OT plays a role in the emergence of maternal behaviors [24].

## 2 Oxytocin transport by RAGE

To exert its function in the brain, OT must be released from oxytocinergic neurons and/or transported into the brain from blood [26,27]. It is well established that OT release in the brain is CD38-dependent [28,29]. Therefore, we will not review the details of CD38-dependent OT release here.

The intranasal route is highly recommended for OT delivery to the brain [[30], [31], [32], [33]]. Although not physiological, it has a therapeutic or pharmacological potential [34]. This approach has been recently used in treatment of autism spectrum disorders and other psychiatric diseases [26,34]. Therefore, transport of OT across the blood-brain barrier is essential for such treatment option [35,36].

In rodents, peripherally administered OT can enter the brain [35,36]. In patients, monitoring of blood flow by magnetic resonance imaging showed that nasally administered OT can be recruited to various brain regions through undefined nasal and other mechanisms. The authors also reported that intranasally administered OT can enter the bloodstream, with subsequent uptake into the brain from the circulation [37]. [35] identified a receptor responsible for transport of OT into the brain, followed by a similar finding in the intestinal barrier of mice [27]. This receptor is specific for advanced glycation end-products (RAGE) [35,38,39]. We reported that RAGE is an OT-binding protein that plays a critical role in OT transfer into the brain from the blood [26,27,35,36,[39], [40], [41]] and into body fluids from the intestine [27].

A reconstituted *in vitro* blood-brain barrier system with cultured monkey vascular endothelial cells has shown evidence of OT transport. In this system, OT was transported predominantly from the luminal (blood) space to the abluminal (brain) space. OT transport in the reverse direction was much lower [35]. In addition, RAGE-dependent and OT-related social memory was further examined using a mouse line with deletion (knockout) of *Ager*, the mouse homologue to the human RAGE gene. We designated this mouse line as RAGE KO mice [35,36,42].

**Fig. 1:**
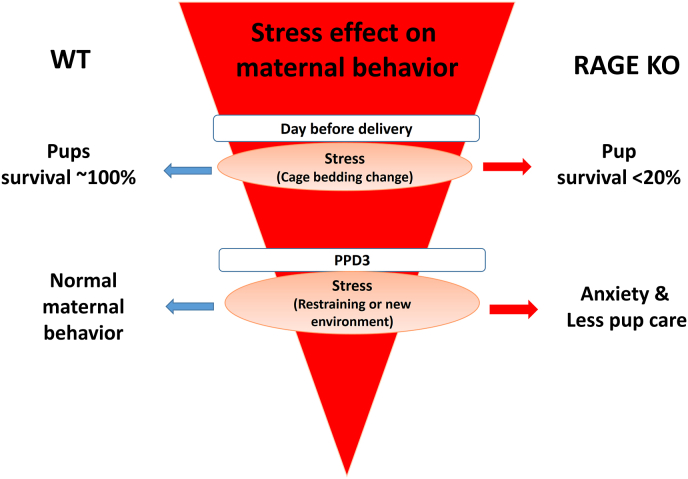
Effect of stress on mice dam behavior. Stress application (cage bedding changing) on the day before delivery resulted in 80% of pups' death in RAGE KO dams but not in WT. Stress on PPD3 (new environment/restraining) led to anxiety and less pup care only in RAGE KO dams.

Male RAGE KO mice are hyperactive [42], while mother mice (dams) exhibit no care for their pups (neglect-like behavior), resulting in death of almost all RAGE KO neonates, when the dams are exposed to social stress (cage switching, referred to as the second hit) one day before delivery (Fig. 1) [35,40]. The KO mothers’ negligent behavior occurred with both KO and wild-type (WT) pups. Therefore, RAGE KO dams display defects in maternal behavior, indicating that RAGE is critical in the puerperium and postpartum period, during which recruitment of OT is mediated by RAGE.

## 3 Child neglect-like behavior

We observed maternal behavioral deficits in RAGE KO mice, which resulted in offspring neglect and low offspring survival (Fig. 1). As offspring of RAGE KO mice transferred to WT postpartum foster mothers were well nurtured and survived at near-normal frequencies, this phenotype is clearly maternal [35]. In contrast, WT offspring was poorly nursed when transferred to postpartum RAGE KO foster mothers. Thus, neglect-like behavior is reminiscent of other disorders related to loss of OT function. This phenotype resembles those of CD38 KO, OT receptor KO, OT KO, CD157 KO, and TRPM2 KO [28,29,[43], [44], [45]]. Although there is some indication that deficits are present also in KO pups, this is largely due to the intrauterine and peripartum effects of loss of OT function in KO dams. This also indicates signaling interactions between OT and RAGE [40].

## 4 Hyperlocomotion and anxiety-like behaviors in the open field after delivery

Locomotor behavior in the open field was tested during the first exposure rather than after habituation, because it indicates environmental (social) stress in mice. The total distance traveled in the whole arena by virgin female WT and RAGE KO mice was not different [40]. However, RAGE KO mothers at postpartum day (PPD) 3 traveled greater distance than virgin female mice. At PPD 7, the distances traveled by both WT and KO dams were equally lower than those of virgin female mice. The time spent in the inner zone, which is an indicator of anxiety-like behavior, was lower in postpartum for both WT and KO dams [40]. These results indicate that the RAGE KO dams at PPD 3 behave abnormally (hyperlocomotion), resembling the behavior of male RAGE KO mice. Interestingly, abnormal behavior in RAGE KO dams mostly disappeared at PPD 7. This suggests that deficits observed at PPD 3 in RAGE KO dams are due to stress, and that these mice partially recover by PPD 7, after the delivery peak stress. In these mice, difference between the early and late postpartum period is clear and may partly resemble the puerperium (early postpartum period) in humans.

## 5 Pup retrieval behavior in the open field

**Fig. 2:**
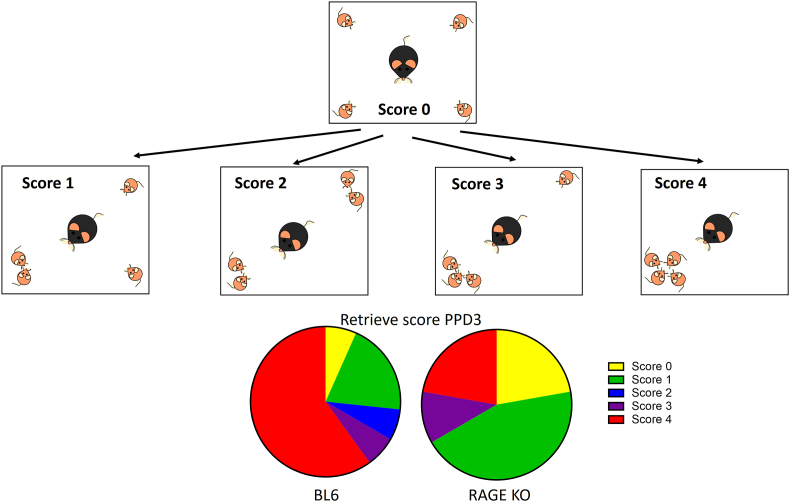
Maternal retrieval behavior of dams at postpartum day (PPD) 3 with pups placed in the four corners of the open field. Scheme of experiments in the open field. Pups were placed at each corner on PPD 3. Scores from 0 to 4 are shown: 0, no retrieval; 1, one pup was retrieved to one of the other corners; 2, two pups were retrieved to two different corners; 3, two pups were retrieved to the same corner; and 4, three pups were retrieved to one corner to join the fourth pup and form a complete nest [40]. Lower graphs indicate maternal scores of wild-type (BL6) or RAGE KO dams at PPD 3. RAGE, receptor for advanced glycation end-products; KO, knockout.

We developed a new experimental paradigm to examine pup retrieval in the open field (Fig. 2 [40]; by modifying a previously reported method [29]. Although RAGE KO mothers displayed abnormal pup care behavior leading to poor pup survival, in the absence of external stress pup care was normal (Fig. 1; [40]. Therefore, the ability of dams to retrieve pups was examined.

In the open field test, WT and RAGE KO dams were first placed in the center of the field (stressed by the exposure to a new environment) with their pups at the four corners. We scored the level of retrieval behavior from 0 to 4 for 10 min, as shown in Fig. 2. A large percentage of maternal scores were 0 or 1 in the RAGE KO dams at PPD 3, while scores were 4 in WT dams at PPD 3.

After introducing the pups in the open field, self-grooming behavior of dams was measured. Interestingly, self-grooming time was longer in RAGE KO than WT dams at both PPDs 3 and 7. Conversely, interaction with the pups was shorter in RAGE KO than WT dams at PPD 3 [40]. These results show that RAGE KO dams were less interested in their pups, indicating a maternal behavior deficit.

## 6 Pup retrieval test after body restraint

Next, we investigated whether stress is necessary for inducing the maternal behavior deficiency in RAGE KO dams. To this end, we applied stress by body restraint for 20 min and observed pup retrieval behavior in WT and RAGE KO mice. Latency in pup retrieval was significantly greater in restrained RAGE KO than WT dams at PPD 3. A similar result was observed in the total time needed to retrieve all five pups [40]. These results indicate that stress results in maternal behavior deficits in KO dams, while under non-stressed conditions the maternal behavior is normal.

## 7 Endogenous or exogenous stress

**Fig. 3:**
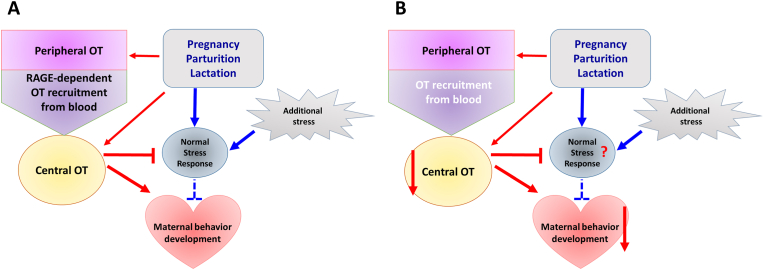
Scheme depicting factors involved in inducing or inhibiting maternal behavior in wild-type dams at postpartum day 3. **(A)** RAGE-dependent oxytocin (OT) recruitment to the brain and facilitation of OT release to the brain by the reproductive processes, including pregnancy, parturition, and lactation, which increase brain (central) OT levels. These OT levels induce maternal behavior. Additional restraint or stressful social stimuli (exogenous stress) activates stress responses (hypothalamus-pituitary-adrenal axis) in mice. These stress signals are also activated by reproductive processes (endogenous stress). However, brain OT levels may be able to reduce (antagonize) the stress response. RAGE, receptor for advanced glycation end-products. **(B)** Stress inputs from additional exogenous stress and reproductive processes are at the same level as for wild-type dams. Because there is no contribution of RAGE to central (brain) OT levels, activation of stress responses is not suppressed. Therefore, signals that inhibit maternal behavior overcome induction signaling from brain OT levels. RAGE, receptor for advanced glycation end-products; KO, knockout; OT, oxytocin.

Inability to suppress the stress response of the hypothalamus-pituitary-adrenal (HPA) axis can result in postpartum mood instability, depression, and impaired maternal behavior [46]. Centrally released OT is known to participate in a wide range of behaviors [47,48] and reduces anxiety by altering the HPA axis [49,50]. Therefore, it is possible that the central OT increase by RAGE in the brain reduces the negative effects of various stresses on maternal behavior (Fig. 3A). This could explain the normal maternal behavior observed in WT dams at PPD 3, even after exposure to stress (Fig. 3A). However, to test this hypothesis, more research is needed. First, what is the central OT concentration upon blocking the RAGE-dependent transport? Second, which brain regions are responsible for dampening HPA activity through RAGE-dependent OT transport. It has not been shown in the neural circuits how RAGE-dependent OT transport impacts its local concentration.

In contrast, abnormal maternal behavior is observed in RAGE KO dams at PPD 3 following induction of stress [40], because lower central OT concentration lessens alteration of the HPA axis (Fig. 3B). The lack of abnormal maternal behavior in KO dams at PPD 7 may be the result of a smaller effect of endogenous stress signals arising from parturition and lactation due to parturition recovery, even if brain OT levels are as low as those of PPD 3. A recent review by Thul et al., reported that plasma OT concentration is not associated with postpartum depression [11], although some reports support this association. Instead, they proposed that OT treatment in patients with postpartum depression is required. In addition, a recent report indicated that intrapartum synthetic OT administration either for labor induction or augmentation is positively but weakly associated with postpartum depression [51], suggesting that withdrawal (decrease) of OT concentrations after childbirth may be associated with post-partum depression.

The HPA axis is suppressed during pregnancy and lactation [46,[52], [53], [54]]. Thus, stress-associated impairment of maternal behavior in RAGE KO dams at PPD 3 can be partly explained by the inability of OT to suppress the HPA axis [52,55].

During the early postpartum period, the HPA axis and OT levels are well balanced under normal conditions, resulting in good pup care (Fig. 3A). However, pregnancy, parturition, and lactation increase brain OT levels [22,56], even if they are the sources of stress (Fig. 3A and B; [57]. On the other hand, the amount of OT produced in the brain is most likely much higher than the amount taken up from the periphery, although the exact levels are unknown. Our hypothesis that peripheral transport to the brain is essential for dampening the stress may need to be reconsidered.

These reproduction-induced stressors (endogenous stress) can be antagonized by brain OT, as previously reported in CD38 KO mice [29]. In these mothers, social stress and HPA axis activity are reduced by social buffering, such as the presence of a mating partner [58].

In the case of *Ager* deletion, stresses suppression is reduced due to absence of RAGE-dependent OT recruitment to the brain (Fig. 3A and B). Next, it is necessary to examine the HPA axis by measuring levels of corticosterone or other stress-responsive factors in RAGE KO mice. In contrast, estrogen withdrawal in Syrian hamsters is involved in postpartum anxiety increase, which is associated with activation of OT neurons in the paraventricular hypothalamus and subsequent activation of OT receptors in the dorsal raphe nucleus [59,60]. It has been suggested that this OT neuroplasticity leads to serotonergic and dopaminergic neurocircuit activation [59]. Therefore, it will be necessary to examine brain region- or circuit-specific mechanisms for induction of postpartum depression via modulation of OT, such as the hypothalamus-raphe circuit.

## 8 Perspective

Future studies should answer currently unsolved questions, such as what causes the main alterations in the maternal behavior of RAGE KO mice, how is RAGE related to the stress, and how RAGE and stress relate to each other when we consider a second hit stressor?

RAGE KO dams can nurse their offspring in the absence of external stress. It remains unknown whether the lack of nursing behavior may cause for the pups to reduce interaction with their mothers, resulting in mother's offspring neglect. To answer this point, it is essential to know if the lack of RAGE also alters the plasma OT level in RAGE KO dams. Furthermore, how is the oxytocin system in mother's brain affected by the RAGE deletion is an important question.

## 9 Conclusion

RAGE is essential during the early postpartum phase and this new hypothesis can be extended to women behavior during the puerperium.

**Fig. 4:**
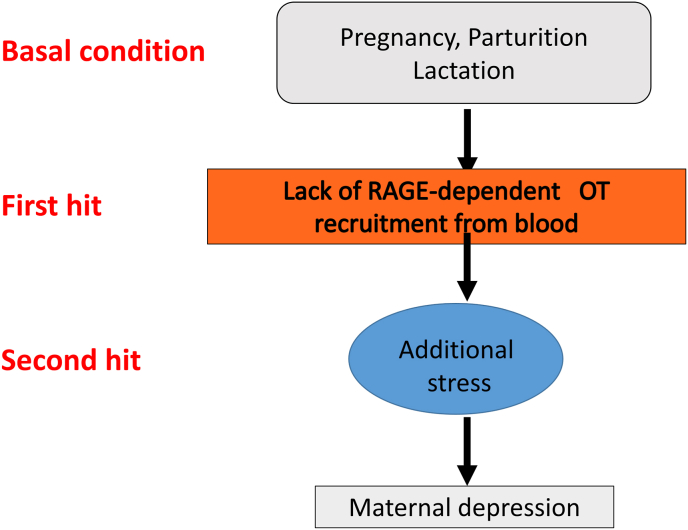
Scheme depicting two hits leading to maternal depression. Dams with reproductive stress conditions (basal condition) will get the first (no RAGE-dependent OT recruitment) and second (exogenous social or restraint stress) hits, resulting in maternal depression. RAGE, receptor for advanced glycation end-products; OT, oxytocin.

When we apply the two-hit theory (two-stage disease model; [61], RAGE impairment may represent the first hit (Fig. 4) for postpartum depression. In the control condition, the RAGE-dependent OT effect may reduce the endogenous stress from reproductive processes. However, any dysregulation or dysfunction of RAGE will trigger the first hit. Exogenous stress, which may represent the second hit, becomes an additional factor leading to maternal behavior deficits. A balanced interaction between RAGE and the HPA is lacking. Mothers with the absence of RAGE-recruited OT do not recognize the need to nurture offspring, leading to anxiety and depression resulting in impaired maternal behavior and neglect of childcare [35,36,40]. Based on data from our mouse studies, it is very important to investigate the relationship between RAGE and human postpartum psychosis, child neglect, anxiety, and depression specifically focusing on genetic, molecular, and neural circuit levels in the future.

There is no data on RAGE involvement in reproductive processes or functions during the reproductive period. Furthermore, there are no reports on gene mutations and single nucleotide polymorphisms related to RAGE associated with depression or psychiatric disorders. However, reports suggest regulatory effects of AGEs on: (i) granulosa cells, adipocyte physiology, obesity and insulin resistance in women with polycystic ovarian syndrome PCOS and in polycystic ovary animal models and (ii) infertility and measures of ovarian reserve [62]. Therefore, genetic analysis on RAGE in women suffering from postpartum depression will be needed.

## Ethics statement

The studies involving mice were reviewed and approved by Ethics Committee for Medical Researches of the Kanazawa University Graduate School of Medical Sciences.

## Author contributions

M.G.,Y.Y. and H.H. designed and performed the behavioral and biochemical experiments. M.G. and H.H. drew figures. M.G.,Y.Y. and H.H. wrote the manuscript.

## Declaration of competing interest

The authors declare that they have no known competing financial interests or personal relationships that could have appeared to influence the work reported in this paper.
